# An Extreme and Successful Case

**Published:** 1872-06

**Authors:** G. D. Billings


### An Extreme and Successful Case.

BY G. D. BILLINGS.

A young lady about twenty-five years of age called at my office on the 7th of November, 1871, with a swollen face, caused from ulceration proceeding from right superior central incisor. Tooth quite sore, and somewhat loose; did not wish to loose it if it could possibly be saved; told her I thought it could be. Inserted a small nerve broach up through nerve canal, but the moment of touching the ulcer she moved so quickly that the instrument was broken off. I worked two hours trying to extract it, but could not succeed. Dismissed patient to call again in two days, with the hope that the broach would come down, so that I could remove it; but when she called, I found that it was further up than when I dismissed her, and she was suffering almost as much pain, but still anxious that the tooth should be saved. I persuaded her to let me extract it, fill, and replace again. It was something new to her, also to me, but at last she consented. I extracted it, removed the broach, syringed with warm water, filled the root and crown with gold, and removed all the ulcer, and syringing the cavity with a solution of carbolic acid and water, replaced it in thirty-two minutes after extracting. Having the tooth back, I began to contrive means to keep it there; I at last settled upon this mode: Placed a rubber band on left superior incisor and carried it (over and under replaced tooth) to the right superior lateral incisor, then placed a small wire on palatine surface of the band and brought it down under the cutting surface of replaced tooth and again attached it to the band on labial surface, with a request to wear it two weeks, which she did. Did not see her again until May 1, 1872, when she called with tooth as nice and solid as any tooth in her mouth. No soreness, and to all appearances will do duty for a good many years yet. Now, if this case should lead some other brother of the profession to try it under such circumstances as I was placed, and he should succeed as well as I did, I will feel amply rewarded.

